# Comparative Analysis of Capsular and Secreted Polysaccharides Produced by *Rhodotorula mucilaginosa* and *Cryptococcus neoformans*

**DOI:** 10.3390/jof9111124

**Published:** 2023-11-20

**Authors:** Gustavo Dornelles, Glauber R. de S. Araújo, Marcus Rodrigues, Vinicius Alves, Rodrigo Almeida-Paes, Susana Frases

**Affiliations:** 1 Laboratório de Biofísica de Fungos, Instituto de Biofísica Carlos Chagas Filho, Universidade Federal do Rio de Janeiro, Rio de Janeiro 21941-902, Brazil; gustavodornelles@biof.ufrj.br (G.D.); glauber@biof.ufrj.br (G.R.d.S.A.); marcusrodrigues@biof.ufrj.br (M.R.); viniciusalves@biof.ufrj.br (V.A.); 2 Laboratório de Micologia, Instituto Nacional de Infectologia Evandro Chagas, Fundação Oswaldo Cruz, Rio de Janeiro 21040-900, Brazil; rodrigo.paes@ini.fiocruz.br; 3 Rede Micologia RJ, Fundação de Amparo à Pesquisa do Estado do Rio de Janeiro (FAPERJ), Rio de Janeiro 21040-360, Brazil

**Keywords:** *Cryptococcus neoformans*, *Rhodotorula mucilaginosa*, fungal virulence, capsule, polysaccharide

## Abstract

Fungal infections are a global public health challenge, especially among immunocompromised patients. *Basidiomycetous* yeasts, such as *Rhodotorula mucilaginosa*, have emerged as opportunistic pathogens, but have received less attention than *Cryptococcus neoformans*. This study aimed to characterize the polysaccharides of *R. mucilaginosa* and compare them with those of *C. neoformans*, analyzing their clinical implications. Comprehensive physicochemical, mechanical, and ultrastructural analyses of polysaccharides from both species were performed, revealing correlations with virulence and pathogenicity. *R. mucilaginosa* cells are surrounded by a capsule smaller than that produced by *C. neoformans*, but with similar polysaccharides. Those polysaccharides are also secreted by *R. mucilaginosa*. Cross-reactivity with *R. mucilaginosa* was observed in a diagnostic *C. neoformans* antigen test, using both *in vitro* and *in vivo* samples, highlighting the need for more reliable tests. Some *R. mucilaginosa* strains exhibited virulence comparable to that of *C. neoformans* in an invertebrate experimental model (*Tenebrio molitor*). This study contributes to a deeper understanding of yeast pathogenicity and virulence, highlighting the need for more accurate diagnostic tests to improve the differential diagnosis of infections caused by basidiomycetous yeasts.

## 1. Introduction

Invasive fungal infections have posed a significant and escalating challenge to public health, particularly among immunocompromised patients. Over the past three decades, there has been a substantial surge in the prevalence of invasive fungal infections, with immunocompromised individuals, intensive care unit (ICU) patients, those with neoplasms, individuals undergoing surgical or invasive procedures, transplant recipients, and notably, patients with acquired immunodeficiency syndrome (AIDS) being particularly vulnerable [1,2,3,4,5]. Additionally, advanced age and extended exposure to antimicrobial therapies are contributing factors to the marked rise in human invasive fungal infections [6]. The recent coronavirus disease-2019 (COVID-19) pandemic, caused by the novel severe acute respiratory syndrome coronavirus 2 (SARS-CoV-2) also became more predisposed to presenting co-infections and superinfections caused by fungi, bacteria, and viruses that are found, making treatment difficult and increasing the risk of morbidity and mortality [7,8,9].

Presently, the survival of transplanted patients, cancer patients, and those with diabetes has rendered them susceptible to opportunistic infections [10]. Furthermore, opportunistic fungi are highly prevalent among people living with HIV/AIDS [11]. In these infections, the weakened host’s immune status plays a pivotal role in the dissemination of these yeasts [12], leading to heightened mortality rates, increased morbidity, and a challenging prognosis [13,14].

Over the past two decades, yeasts have gained recognition as significant opportunistic pathogens within clinical settings [15]. While *Cryptococcus* spp. infections have garnered substantial attention and research, infections caused by *Rhodotorula* spp. warrant greater focus and investigation, as these yeasts have not been traditionally highlighted as primary potential agents causing fungal diseases. Nevertheless, recent advancements in research and microbiological studies have revealed a growing prevalence of this yeast in fungal infections. Additionally, *Rhodotorula* spp. share common characteristics with *Cryptococcus* spp., which can lead to erroneous diagnoses and inappropriate patient treatment [16].

*Rhodotorula* species are basidiomycetous yeasts and have a close relationship with the *Sporidiobolaceae* family. These yeasts are distributed widely across various natural environments [17]. The *Rhodotorula* genus yields colonies with pinkish to red hues [18], characterized by unicellular blastoconidia and the absence of pseudohyphae and hyphae. Researchers have isolated *Rhodotorula* from diverse environmental sources and identified infections in animals [12]. This cosmopolitan fungus thrives in biomes worldwide, even in colder regions such as polar and subpolar areas on Earth [19].

In 1967, Louria et al. [20] conducted studies on mice, revealing that fungal agents of the *Rhodotorula* genus exhibited limited ability to persist and multiply within murine tissues, even in animals immunosuppressed with corticosteroids, as compared to *Candida* spp. infections [20]. Interestingly, no cases of *Rhodotorula* infection were reported in the medical literature until 1985, after which the incidence of infections grew, potentially coinciding with advances in medicine and the increased utilization of intensive care and central venous catheters [12]. Within the last two decades, *Rhodotorula* spp. have gained recognition as emerging pathogens in humans [21]. Up to 2011, a total of forty-seven distinct *Rhodotorula* species were described, showcasing extensive taxonomy [21]. And with the increase in these fungal infections considered uncommon or rare, due to the increase in immunocompromised patients in recent years, *Rhodotorula* yeast is among the main emerging fungal pathogens [14,22]. Among these, *R. mucilaginosa*, *R. glutinis*, and *R. draft* are the most prominent culprits causing disease in humans [23]. Among *Rhodotorula* species, the species *R. mucilaginosa* is the most common cause of fungemia, causing up to 79% of infections, followed by *R. glutinis* (7.7%) [24]. Researchers have continued to isolate *Rhodotorula* from diverse environments and document infections in animals [12]. A case of meningitis caused by *R. mucilaginosa* in an individual living with HIV/AIDS was reported in 2008 by Baradkar and Kumar [25]. *Rhodotorula* species have been implicated in causing septicemia, meningitis, endocarditis, keratitis, ventriculitis, peritonitis, central venous catheter infections, and ophthalmic infections [25,26,27,28,29,30,31,32,33,34]. While *Rhodotorula* spp. exhibit a lower prevalence of fungal endocarditis compared to other fungi with greater infectious potential, such as *Candida* spp., *Aspergillus* spp., and *Histoplasma capsulatum*, it is crucial to consider *Rhodotorula* spp. as high-risk pathogens for infective endocarditis in immunosuppressed individuals and those with central venous catheters [32,35,36]. According to Almeida et al. (2008), the progression of infection is severe, with a lethality rate ranging from 12% to 20% [37]. In countries such as China, the *Rhodotorula* genus is among the main causes of invasive fungal infections caused by non-candida yeasts [38].

*Rhodotorula* spp. and *Cryptococcus* spp. share several characteristics, such as their ability to assimilate urea and their inability to ferment carbohydrates, contributing to their similar morphologies. However, fungi within the *Cryptococcus* genus do not produce the carotenoid pigments present in *Rhodotorula* spp., which do not assimilate inositol or produce melanin, unlike *Cryptococcus* spp. [39,40]. The most crucial virulence factor in *C. neoformans* is the polysaccharide capsule [41,42]. Yockey et al. (2019) [16] conducted a capsule size comparison between environmental and clinical strains of *R. mucilaginosa*, revealing that clinical strains yield capsules with larger dimensions than those of environmental origin. However, knowledge concerning its pathogenic effects and functions remains limited [43]. Overall, *R. mucilaginosa* capsules were smaller than those of *C. neoformans* [16]. Differences in structure and composition have led to the use of polysaccharides found in fungal cell walls as crucial markers for taxonomical classification of different fungal species [44,45,46,47].

Polysaccharides, specifically (1→3)-β-D-glucan, present in the cell wall of *Rhodotorula* spp., have been employed as important markers for the taxonomical classification of different fungal species [44,45,46,47]. The presence of these polysaccharides has significant implications, but limited information is available about the structural and secreted polysaccharides produced by *Rhodotorula* spp. Garza et al. (2016) analyzed the secreted polysaccharides of *R. mucilaginosa* under stress conditions, such as acidic pH, aeration, and exposure to heavy metals. They achieved promising results in the production of exopolysaccharides, suggesting its potential role in environmental bioremediation [48].

Given the compositional similarities in the capsular polysaccharides of *Rhodotorula* and *Cryptococcus*, the main objective of this study is to conduct a comprehensive investigation and comparative analysis of the shared and distinctive characteristics displayed by *Rhodotorula* spp. and *Cryptococcus* spp., with a specific focus on their polysaccharide capsule attributes. By unveiling both the commonalities and distinctions in these fundamental aspects of these two fungal genera, this research aims to provide valuable insights into their diagnosis and potential implications for pathogenicity.

## 2. Materials and Methods

### 2.1. Strains

Environmental (SA1, PO1, VG4) [49] and clinical (RC1) *Rhodotorula* strains were obtained from Coleção de Fungos Patogênicos (Pathogenic Fungi Collection) at the Instituto Nacional de Infectologia Evandro Chagas, Fiocruz, RJ, Brazil [49,50]. For comparison, the standard strain of *C. neoformans type strain H99* (available through the American Type Culture Collection catalog number 208821), which was originally isolated in February 1978 by Dr. John Perfect at Duke University Medical Center from a 28-year-old male with Hodgkin’s disease, was used as a control. All strains isolated were securely stored at a temperature of −80 °C.

### 2.2. Inducing Formation, Visualization, and Measurement of Yeast Capsular Size by Optical Microscopy and Contrast with India Ink in Isolates

Polysaccharide capsule production was performed in a nutrient-deprived medium known as Minimal Medium (MM). This medium consists of 15 mM glucose (Cat# G5767), 10 mM MgSO_4_·7·H_2_O (Cat# 230391), 29 mM KH_2_PO_4_ (Cat# P0662), 13 mM glycine (Cat# 50046), and 3 µM thiamine (Cat#T4625) with pH 5.5 ± 0.1 at 25 °C—(all components Merck Millipore, Burlington, MA, USA). Culture times varied to promote gradual polysaccharide production on specific experiments. The cultures were incubated at 36.5 °C (±0.5 °C).

For microscopy analyses, a suspension of 1 × 10^6^ cells/mL was centrifuged at 6708× *g* for 5 min and washed once with PBS (pH 7.2 ± 0.2 to 25 °C) (Cat# P4417, Sigma-Aldrich, Saint Louis, MO, USA). To perform morphometry and increase contrast, the yeasts were contrasted with Indian ink. Slides were prepared with 5 µL (5 × 10^3^ cells counting in a Neubauer chamber) of the suspension containing the fungal cells and 5 µL of the Indian ink, and visualized in an optical microscope (AXIO Lab.A1, Zeiss, Jena, Germany). A systematic approach was taken to capture random images, ensuring the inclusion of a minimum of 100 cells per analysis. The images were subsequently subjected to analysis using the ImageJ 1.53t bundled with Java 1.8.0_345 (64 bits) for macOS 13 (Ventura) software (http://rsb.info.nih.gov/ij/, accessed on 7 July 2023) provided by the National Institutes of Health (NIH, Bethesda, MD, USA). The software facilitated the measurements of both capsule length and cell body dimensions.

### 2.3. Isolation of Secreted Polysaccharides from Yeast Culture Supernatant via Ultrafiltration

The accumulated secreted polysaccharides (PS) present in the culture supernatants were isolated using the Amicon^®^ stirred cells (Millipore, Danvers, MA, USA). A membrane with a molecular weight cutoff of 10 kDa was employed in the ultrafiltration process to recover the polysaccharides. The quantification of the final solution was accomplished using the phenol-sulfuric colorimetric method, with glucose serving as the standard [51].

### 2.4. Determining the Effective Diameter and Hydrodynamic Radius of PS Samples Using Dynamic Light Scattering (DLS)

The PS samples underwent comprehensive analysis utilizing the NanoBrook Omni particle equipment, sourced from Brookhaven Instruments Corporation in Holtsville, NY, USA. This technique entails establishing a correlation between the size and distribution of various particles based on the intensity of light scattered due to the Brownian motion of suspended particles. The measurements were meticulously conducted at a temperature of ~25 °C. The assessment of effective diameter and polydispersion within the isolated PS was conducted using dynamic light scattering techniques.

The Stokes–Einstein equation then gives the hydrodynamic radius, *R_h_*, corresponding to the measured $R_{h}=\frac{\kappa T}{6\pi \eta Dt}$ where *k* is Boltzmann’s constant, *T* is the temperature in *K*, and *η* is the solvent viscosity. This method capitalizes on the dynamic signal originating from the Brownian motion of PS particles within a liquid suspension, coupled with the resultant variations in scattered light intensity over time. In DLS, the fluctuations in light intensity measured over time are quantified via a second-order correlation function $g^{\left(2\right)}\;\left(\tau \right)$. The autocorrelation function of intensity is shifted by a delay time (*τ*), and the autocorrelation function *g*(*τ*) is calculated $g^{\left(2\right)}\;\left(\tau \right)=1+\beta \exp\left(-2\Gamma \tau \right)$, where *β* is the correlation function amplitude at zero delay, Γ is the decay rate, and the baseline of the correlation function relaxes to a value of 1 at infinite delay.

A nonlinear least square fitting algorithm was used to fit the measured correlation function decay rate Γ. Considering that Γ is associated with relaxation fluctuations, it can be converted to the translational diffusion coefficient Dt for the particle via the following relation: $Dt=\frac{\Gamma }{q^{2}}$. The value of q was computed from parameters including the scattering angle *θ*, the laser light wavelength λ_0_, and the refractive index *η* of the solvent suspension. This was performed using the equation $q=\frac{4\pi n}{\lambda 0}\;Sin\frac{\theta }{2}$.

For extracting the particle size distribution from light scattering data, the non-negative least squares algorithm (NNLS) was employed. This algorithm excels in identifying multimodal size distributions, which are characteristic of samples containing particles of varying sizes. The insights derived from the algorithm-generated data offer a deep understanding of sample polydispersion, a metric that gauges the extent of particle size variation within a given sample.

### 2.5. Characterization of Zeta Potential (ζ) and Conductance of Polysaccharides from Rhodotorula and Cryptococcus Using DLS

Based on the principles of the dynamic light scattering technique (DLS) via the NanoBrook Omni particle instrument from Brookhaven Instruments Corporation in Holtsville, NY, USA, we conducted an analysis of two crucial electrokinetic properties: phase analysis light scattering (PALS) to determine the electrophoretic mobility of charged, colloidal suspensions denominated Zeta potential (ζ), and cell conductance.

The Zeta potential (ζ) can be calculated using the Henry equation, which relates that the electrophoretic mobility (μ) is defined as the potential at the plane where the liquid velocity relative to the particle is zero. This plane, which is called the slipping plane (or the shear plane), does not necessarily coincide with the particle surface. Only if the slipping plane is located at the particle surface, the zeta potential becomes equal to the surface potential (measured in millivolts) situated on the surface of particles suspended within a liquid medium. The zeta potential (in mV) is defined by the equation $\zeta =\frac{\varepsilon \;\eta \;\mu }{2\varepsilon 0}$ where ε is the dielectric constant of the dispersing medium, H is the viscosity of the dispersing medium (in cP or mPa·s), μ is the electrophoretic mobility (in μm/s per V/cm), and ε_0_ is the vacuum permittivity (approximately 8.854 × 10^−12^ C^2^/N·m^2^). This metric is significantly impacted by diverse factors, including pH, ionic strength, ion concentration, and temperature, among others. The determination of the Zeta potential bears vital importance in comprehending the stability of suspended particles, given its association with the electrostatic forces governing particle interactions.

On the other hand, conductance stands as a measurement gauging the capacity of particles to conduct electricity when subjected to an electric field. Conductance is susceptible to influences from various aspects, encompassing the concentration and nature of ions present within the solution, as well as the particle size. For measurement purposes, the samples were prepared using a 10 mg/mL stock of concentrated polysaccharides acquired through ultrafiltration. Specifically, 50 µL of this polysaccharide stock solution was blended with 1.5 mL of apyrogenic water within suitable polystyrene cuvettes.

### 2.6. Characterization of Passive Microrheology (µRh) of Polysaccharides from Rhodotorula and Cryptococcus Using DLS

Passive microrheology is a technique used to study the mechanical properties and rheological behavior of complex fluids, such as colloidal suspensions, polymer solutions, and biological fluids. This method relies on tracking the thermally induced motion of a submicrometer-sized probe suspended within the fluid. Unlike active microrheology, which exerts external forces on the probe to stimulate motion, passive microrheology remains nonintrusive, preserving the innate state of the material under investigation. The fundamental principle of passive microrheology centers on gauging the probe’s diffusion through the viscoelastic medium it occupies. This diffusion-derived probe motion yields insights into the material’s mechanical traits, encompassing the viscous (G″) and elastic (G’) moduli, alongside the complex viscosity (η*)—a composite metric embracing both the viscous and the elastic modulus [52].

Passive microrheology, as described, furnishes an avenue to comprehending the intricate viscoelastic dynamics of polysaccharides sourced from *Rhodotorula* and *Cryptococcus*, without imposing any perturbations on their inherent characteristics. For conducting passive microrheology, we harnessed the NanoBrook Omni particle instrument by Brookhaven Instruments Corporation (Holtsville, NY, USA). The setup featured a measurement chamber hosting secreted PS from *Cryptococcus* spp. and *Rhodotorula* spp. under diverse experimental conditions, in conjunction with submicrometric probes (1.00 ± 0.1 μm Polybead^®^ Cat. # 07310-15, Polysciences, Inc., Warrington, PA, USA). This chamber was diligently maintained at a consistent temperature, and measurements were diligently executed in a controlled environment to nullify external interferences. Throughout the measurement process, the probe was illuminated by a light beam, and its position meticulously tracked via a position detection system. The root mean square displacement (MSD) of the probe, indicative of its diffusion within the viscoelastic medium, was computed from its movement. From these MSD data, the viscoelastic properties of the scrutinized material were derived.

### 2.7. Comparative Analysis of Isolated Polysaccharides from Rhodotorula and Cryptococcus Using the CrAg Lateral Flow Assay (CrAg-LFA)

In this study, we employed the CrAg lateral flow assay (CrAg-LFA, IMMY, Norman, OK, USA) to conduct an immunologic similarity analysis on the isolated polysaccharides from *Rhodotorula* and *Cryptococcus*. Our approach involved both qualitative and semiquantitative procedures to discern the characteristics of these polysaccharides. For the qualitative procedure, isolated polysaccharides were prepared at a concentration of 1 mg/mL and subsequently diluted 1:2 [V:V] in 1× sample diluent. In the semiquantitative procedure, samples underwent a 1:5 [V:V] dilution in 1× sample diluent, followed by serial 1:2 [V:V] dilutions. These dilutions were then subjected to the same analysis procedure as employed in the qualitative approach. The experimental process entailed placing the prepared samples in suitable reservoirs, such as test tubes or microtiter plates, while aligning the flow device side with the reservoir. This setup enabled the sample to contact the test membrane, thus facilitating evaluation.

For an *in vitro* assay, fungal cells were cultured in liquid Sabouraud medium for 24 h at 36.5 °C (±0.5 °C) under constant agitation (approximately 100 rpm~5.59× *g*). Subsequently, the cells were centrifuged, and minimal medium (1 mL) was introduced. The cultures were incubated for 3 days at 36.5 °C (±0.5 °C) under continuous agitation as above, fostering the production of both capsular and secreted polysaccharides. The resultant cultures were divided into three categories: (I) cells with supernatants; (II) supernatants only; and (III) concentrated polysaccharides (10 mg/mL) acquired through Amicon^®^ stirred cells (Millipore, Danvers, MA, USA). In our analysis, fungal cell concentrations of 10^4^ cells/mL were employed. For the extraction of concentrated polysaccharides using the Amicon™ Bioseparations Stirred Cells with a cutoff of 10 kDa (Cat # 5123—item discontinued—Millipore, Danvers, MA, USA) operating pressure 30 psi (2.06843 bar), we followed a 10 μL of polysaccharide mixed with 10 μL of diluent and 180 μL of ultrapure water (UltraPure™ DNase/RNase-Free, Cat # 10977015, Thermo Fisher Scientific, Waltham, MA, USA) ratio. For the control assessments, we utilized the following: the manufacturer-provided positive control, the manufacturer-provided negative control (diluent), and a negative control using minimal medium. These controls served as benchmarks against which the experimental results were compared.

For the *in vivo* test, 6–8-week-old female of Class: Mammalia, Order: *Rodentia*, Family: *Muridae*, Genus: *Mus*, Species: *Mus musculus*, (Bagg’s albino) BALB/c mice, with body mass 26.0 ± 3 g, and with food and water *ad libitum*, infected intranasally with an inoculum of 1 × 10^6^ *R. mucilaginosa* cells/mL were used. The inoculum was prepared with a Neubauer chamber. As a control group, mice were infected with *C. neoformans* H99 or with sterile PBS buffer. Eight animals were infected in each group. After a 7-day period, blood samples were collected intracardially, then centrifuged at 6708× *g* for 10 min, and the resulting serum was employed in the CrAg test, following the procedure described earlier. All animals involved in this project received treatment in compliance with current legislation, as overseen by ethics committees responsible for the management of experimental animals (ref: 112/17).

### 2.8. Immunofluorescence with 18B7 Antibody and Chitin Visualization with Uvitex 2B

Yeast cells (1 × 10^6/^mL counting in a Neubauer chamber) were pelleted by centrifugation at 6708× *g* for 5 min at 25 °C, and subsequently resuspended in phosphate-buffered saline (PBS) (pH 7.2 ± 0.2 at 25 °C) containing paraformaldehyde 4% aqueous solution, EM Grade (Electron Microscopy Sciences, Hatfield, PA, USA). The cells were incubated at room temperature (~25 °C) for 30 min. After fixation, the yeast cells were washed twice with PBS, and then incubated in 1% bovine serum albumin (BSA) (Cat# A7906, Sigma-Aldrich, Saint Louis, MO, USA) in PBS (PBS-BSA) for 1 h at room temperature. Following another round of washing, the cells were incubated for 1 h at room temperature in the presence of mAb 18B7 (10 µg/mL), a mouse IgG1 monoclonal antibody known for its high affinity for GXM of distinct cryptococcal serotypes [53]. Uvitex 2B (Ex_max_ 350 nm and Em_max_ 435 nm in PBS) (Cat# 19517 Polysciences, Inc., Warrington, PA, USA), which binds to cell wall chitin and chitosan, was employed in the subsequent steps. After washing in PBS, the cells of interest were treated with 0.1 mg/mL Uvitex 2B for 20 min at room temperature. The cell suspensions were adhered to 18 × 18 mm #1 cover slips (Knittel Glasbearbeitungs GmbH, Bielefeld, Germany), coated with 0.01% poly-L-lysine (Cat#P4832, Sigma-Aldrich, Saint Louis, MO, USA). These prepared slides were then mounted on 26 × 76 mm glass cover slips with a thickness of 1 (Knittel Glasbearbeitungs GmbH, Bielefeld, Germany) for analysis. Fluorescence microscopy was conducted using a Elyra PS.1 microscope (Carl Zeiss Microscopy, Jena, Germany). The resulting images were processed using ImageJ 1.53t bundled with Java 1.8.0_345 (64 bits) for macOS 13 (Ventura) software (http://rsb.info.nih.gov/ij/, accessed on 7 July 2023).

### 2.9. Survival Experiments in Tenebrio molitor Model

In the present study larvae of the Class: *Insecta*, Order: *Coleoptera*, Infraorder: *Cucujiformia*, Family: *Tenebrionidae*, Genus: *Tenebrio*, Species: *Tenebrio molitor* were carefully selected based on their size and the absence of pigmentation marks to ensure consistent and replicable results. The chosen larvae were subjected to inoculation with a 10 µL suspension of yeasts (10^6^ cells counting in a Neubauer chamber) from different strains. The inoculation was performed through an injection into the last left proleg, using a sterile insulin syringe equipped with a 26G gauge needle (0.45 × 13 mm). Prior to injection, the proleg area was cleansed using a cotton swab soaked in 70% ethanol. Following injection, the larvae were placed onto 90 mm glass plates and subsequently incubated at temperatures of 25 °C and 37 °C. The number of deceased larvae was recorded daily. The experimental groups (10 larvae per group) were as follows: (1) sham group (no treatment or manipulation, serving as a negative control); (2) inoculated with phosphate-buffered saline (PBS) (pH 7.2 ± 0.2 to 25 °C) (negative control); and (3) infected with the various yeast strains under study. Each experiment was replicated at least twice (duplicates) across different time periods.

### 2.10. Statistical Analysis

Statistical analysis was performed using GraphPad Prism version 9.5 (GraphPad Software, San Diego, CA, USA) for Windows 11 or macOS Ventura (version 13). The morphometric and physicochemical data underwent statistical analysis using the Student’s *t*-test. The survival rate differences among different *T. molitor* groups were assessed using the log-rank test (Mantel-Cox). The *p*-values for multiple comparisons were calculated through analysis of variance (ANOVA) and adjusted utilizing Tukey’s multiple comparison test. A significance level of *p* < 0.05 was employed.

## 3. Results

### 3.1. Comparative Structural and Physicochemical Analysis between C. neoformans and R. mucilaginosa Cells

**Figure 1:**
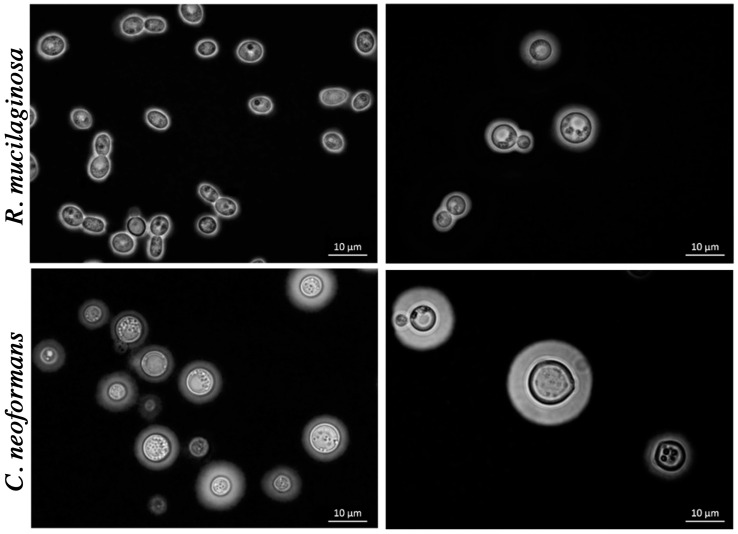
Yeast cells contrasted with India ink. In the upper panels, images of *R. mucilaginosa* yeast strains PO1 and VG4 are displayed, respectively. The presence of a distinct halo surrounding the cell body is evident, indicating the presence of the polysaccharide capsule. The bottom panels show rounded *C. neoformans* cells (H99), serving as a control, where the noticeable presence of a polysaccharide capsule around the cell body is observed.

To conduct a comprehensive comparative analysis encompassing the structural, physicochemical, and biological attributes of *R. mucilaginosa* and *C. neoformans*, we utilized four strains of *R. mucilaginosa*. These strains were compared with the reference strain of *C. neoformans*, H99. Our investigation revealed the presence of a polysaccharide capsule in both yeast species. This feature was identified through the manifestation of a refractive halo, characterized by a distinct white halo that forms around the cell body. The yeast cells of *R. mucilaginosa* strains and *C. neoformans* H99 were subjected to negative-staining using India ink. They were subsequently examined under an optical microscope to facilitate the measurement of both the cell body and the polysaccharide capsule (Figure 1).

**Figure 2:**
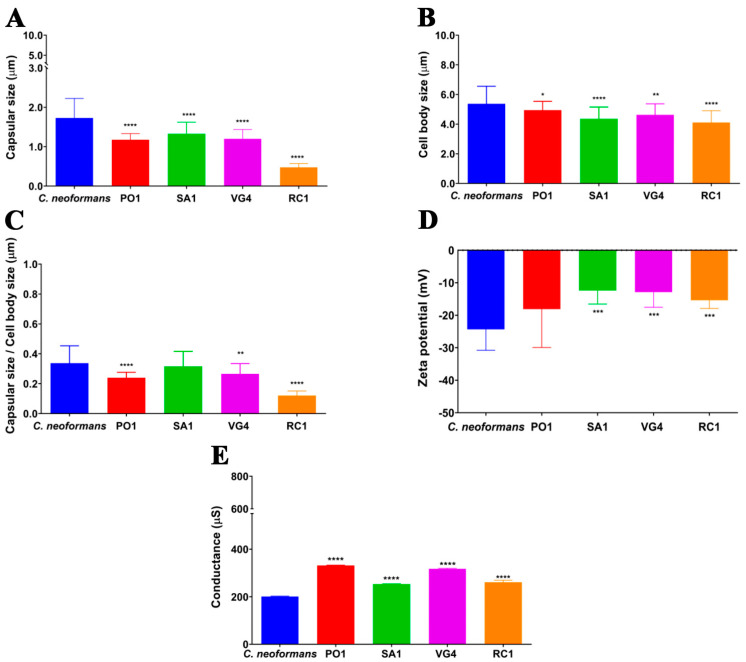
Comparative morphometric and structural analysis of *C. neoformans* (H99) and *R. mucilaginosa* cells (PO1, SA1, VG4, and RC1). (**A**) Capsular size in micrometers, (**B**) cell body in micrometers, (**C**) ratio of capsular size to cell body size expressed in micrometers, (**D**) Zeta potential expressed in millivolts, and (**E**) conductance expressed in microsiemens. Results are expressed as the mean and standard deviations of 100 measurements. **** (*p*-value < 0.00001); *** (*p*-value < 0.0001); ** (*p*-value < 0.001); * (*p*-value < 0.01) by *t*-test.

Notably, the thickness of the capsule and the cell body exhibited significant variation among the different strains of *R. mucilaginosa* and the *C. neoformans* strain. The analysis of the capsule size in both species based on optical microscopy images shows a significantly larger capsule size in the yeast cells of *C. neoformans* compared to the *R. mucilaginosa* strains. In addition to differences in capsular size, both species exhibit statistically significant variations in cell body size. Specifically, the yeast cells of *C. neoformans* are notably larger in size compared to the analyzed *R. mucilaginosa* strains, as illustrated in Figure 2. Based on these findings, we conducted an analysis to explore the correlation between capsular size and cell body size among the studied strains. Notably, one *R. mucilaginosa* strain (SA1) exhibited parity with the *C. neoformans* strain in terms of these measurements. In contrast, the remaining two *R. mucilaginosa* strains (PO1 and VG4) displayed a significant decrease in cell body diameter. This discrepancy prompts us to consider that the SA1 strain, in terms of its capsule-to-cell body ratio, might possess higher virulence potential compared to the other two *R. mucilaginosa* strains. This inference arises from the greater resemblance of the SA1 strain to the *C. neoformans* strain, as depicted in Figure 2.

The Zeta potential (ζ) of the cells was an additional aspect examined, involving a comparison between the control strain of *C. neoformans* and the *R. mucilaginosa* strains PO1, SA1, RC1, and VG4. The observations revealed that *C. neoformans* displayed a notably more electronegative Zeta potential in contrast to the SA1, VG4 and RC1 strains of *R. mucilaginosa*. Interestingly, when assessing the PO1 strain of *R. mucilaginosa*, no substantial disparity was evident when compared to the *C. neoformans* strain (as illustrated in Figure 2). Regarding conductance, a statistically significant distinction emerged among all *R. mucilaginosa* strains when contrasted with the *C. neoformans* strain (as depicted in Figure 2). Notably, the *R. mucilaginosa* strains exhibited markedly higher conductance than the *C. neoformans* H99 strain. This discrepancy suggests an elevation in the presence of ionic charges under these conditions.

### 3.2. Comparative Structural and Physicochemical Analysis of Secreted Polysaccharides from C. neoformans and R. mucilaginosa

**Figure 3:**
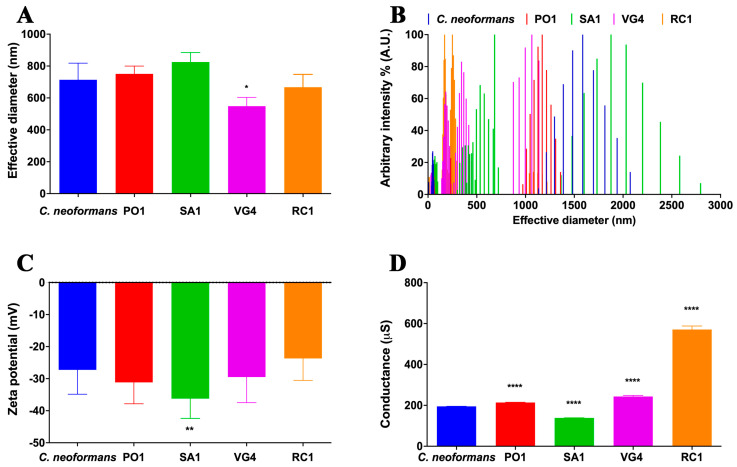
Comparative structural analysis of *C. neoformans* (H99) and *R. mucilaginosa* (PO1, SA1, VG4, and RC1) secreted polysaccharides. (**A**) Effective diameter expressed in nanometers; (**B**) effective diameter presented by intensity (arbitrary unit), highlighting the different size populations of the different strains; (**C**) Zeta potential expressed in millivolts; and (**D**) conductance expressed in microsiemens. **** (*p*-value < 0.00001); ** (*p*-value < 0.001); * (*p*-value < 0.01) by *t*-test.

To explore variations in the properties of PS, an analysis of their size distribution intensity was conducted (Figure 3). By evaluating the averages, it becomes apparent that *C. neoformans* exhibits heightened intensity in the effective diameter of its polysaccharides, primarily ranging from approximately 1500 to 2000 nm. Conversely, *R. mucilaginosa* strains displayed distinct patterns. For instance, the PO1 strain’s polysaccharides exhibited effective diameters within the range of 1000 to 1500 nm. On the other hand, the SA1 strain showcased polysaccharides with an effective diameter split into two prominent intensity clusters: the first cluster featured PS sizes around 500 nm, while the second displayed PS sizes of approximately 2000 nm. Similarly, the VG4 strain also demonstrated polysaccharides of notable intensity with varying effective diameters, ranging from 200 to 500 nm, along with another subgroup of slightly larger PS with an average of 1000 nm, while the RC1 strain presented PS with an effective diameter divided into two groups, one with high intensity presenting sizes around 250 to 300 nm and one with low intensity presenting sizes of 1000 nm.

Regarding the Zeta potential of the secreted polysaccharides, it is noteworthy that only the *R. mucilaginosa* SA1 strain displayed a significant difference in comparison to that of *C. neoformans*. This strain exhibited a higher level of electronegativity compared to the other samples under study (as illustrated in Figure 3). On the other hand, upon examining the conductance of the polysaccharides, we observed substantial variations across all *R. mucilaginosa* strains in contrast to the *C. neoformans* strain. These differences were evident in the conductance measurements, as depicted in Figure 3.

**Figure 4:**
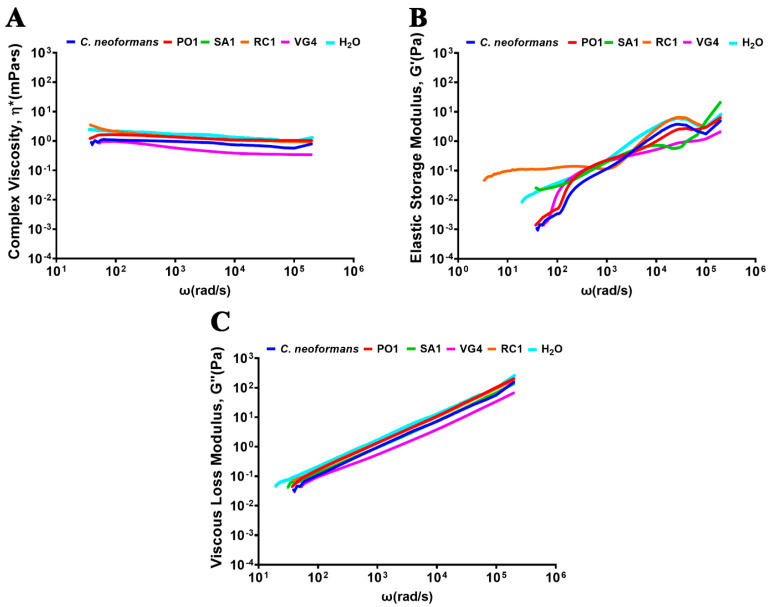
Comparative rheological analysis. (**A**) Complex viscosity, (**B**) elastic modulus, and (**C**) viscous modulus of secreted polysaccharides obtained from various strains, including *R. mucilaginosa* (PO1, SA1, VG4, and RC1) and *C. neoformans* (H99).

In an effort to compare potential alterations in the viscoelastic properties of these PS, we employed the passive microrheology technique. This technique employs micrometric probes to acquire results for complex viscosity (ƞ*), elastic modulus (G′), and viscous modulus (G″). The outcomes yielded insights into the viscoelastic behavior at both low and high frequencies. Enticingly, the findings revealed that there were no significant alterations in the viscous complex for most strains. However, the RC1 strain did exhibit a slight discrepancy at low frequencies when compared to the other strains. Conversely, the SA1 and PO1 strains displayed such similar characteristics that their curves overlapped (as depicted in Figure 4). Similar observations were made during the analysis of the elastic complex structure. Except for the *R. mucilaginosa* strain RC1 at low frequencies, the strains did not remarkably diverge in terms of their elastic behavior (refer to Figure 4). Furthermore, the structural analysis of the viscous modulus did not highlight notable variations or differences among the strains (as shown in Figure 4).

### 3.3. Comparative Analysis of Antigenic Determinants between C. neoformans and R. mucilaginosa

**Figure 5:**
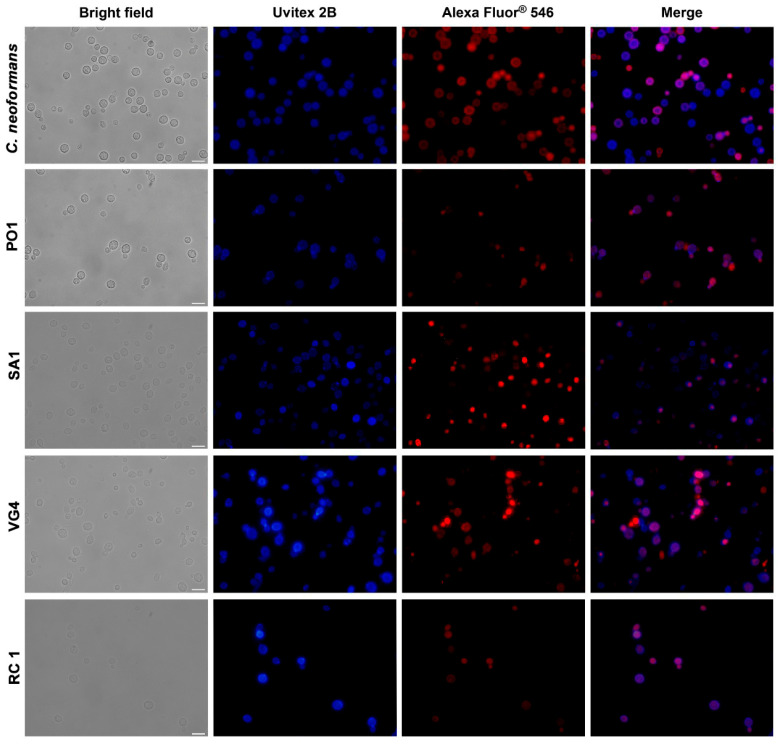
Immunofluorescence of *C. neoformans* and *R. mucilaginosa* cells using 18B7 (in red, a monoclonal antibody targeting polysaccharide capsule epitopes) and Uvitex 2B (in blue, for chitin cell wall staining). Bars: 10 µm.

For the comparative analysis of antigenic determinants between *C. neoformans* and *R. mucilaginosa* strains, we employed immunolabeling with 18B7, an antibody against epitopes of the capsular polysaccharide of *Cryptococcus*. All strains displayed specific labeling with the 18B7 antibody, suggesting the presence of similar epitopes in the polysaccharide capsules of these *R. mucilaginosa* yeasts (Figure 5).

Given the significant similarity in physicochemical properties and immunolabeling characteristics between these genera, we explored the potential for cross-reactivity between the rapid test designed for *Cryptococcus* spp., known as the cryptococcal antigen lateral flow assay (CrAg-LFA), and *R. mucilaginosa*.

**Figure 6:**
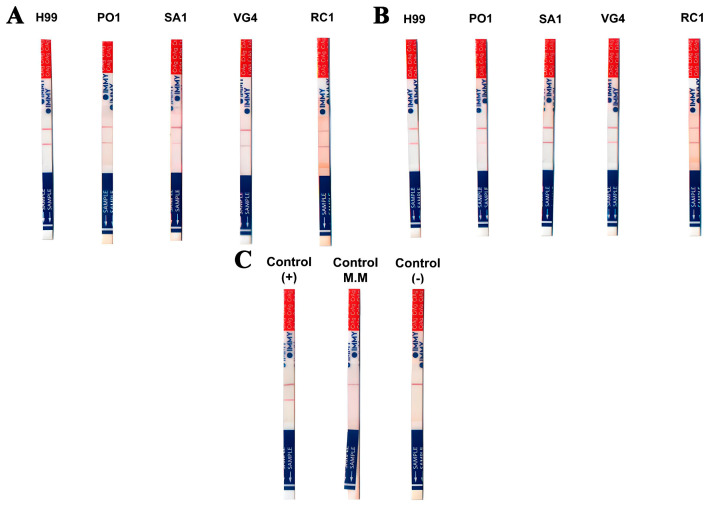
*In vitro* CrAg-LFA analysis. (**A**) Testing performed using cells and culture supernatants. (**B**) Outcomes derived from the secreted polysaccharides of all tested samples. (**C**) Positive and negative control groups.

To investigate this possibility, we initially conducted an *in vitro* test using *R. mucilaginosa* strains in conjunction with the reference strain of *C. neoformans*. Initially, we analyzed the total sample, comprising both cells and supernatant, and observed positive results in all tested samples, indicating *in vitro* cross-reactivity with the rapid cryptococcal antigen detection test (Figure 6). We also conducted tests on samples containing only the supernatant and secreted PS, which yielded positive results in all five fungal strain samples, thus demonstrating cross-reactivity between *Rhodotorula*’s secreted polysaccharides and cryptococcal GXM (Figure 6).

**Figure 7:**
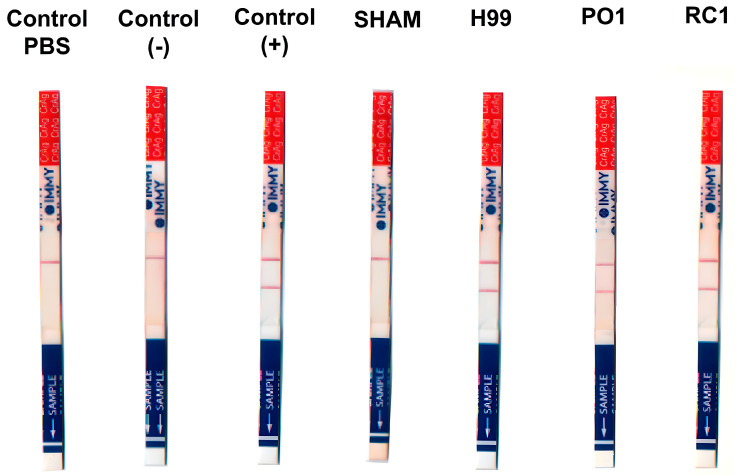
*In vivo* CrAg-LFA analysis. Sera collected from animals infected with *R. mucilaginosa* strains and *C. neoformans*, and corresponding positive and negative controls from infected and uninfected animals. Analysis of CrAg-LFA *in vivo*: Sera obtained from animals infected with *R. mucilaginosa* strains and *C. neoformans*, along with positive and negative controls from both infected and uninfected animals.

Following the *in vitro* results, we extended the cross-reactivity experiments to *in vivo* studies using Balb/c mice. All animals infected with *Rhodotorula* strains tested positive for the CrAg test. This finding highlights the existence of cross-reactivity between the secreted polysaccharides of *Rhodotorula* and cryptococcal GXM (Figure 7).

### 3.4. Virulence Analysis in Tenebrio molitor

**Figure 8:**
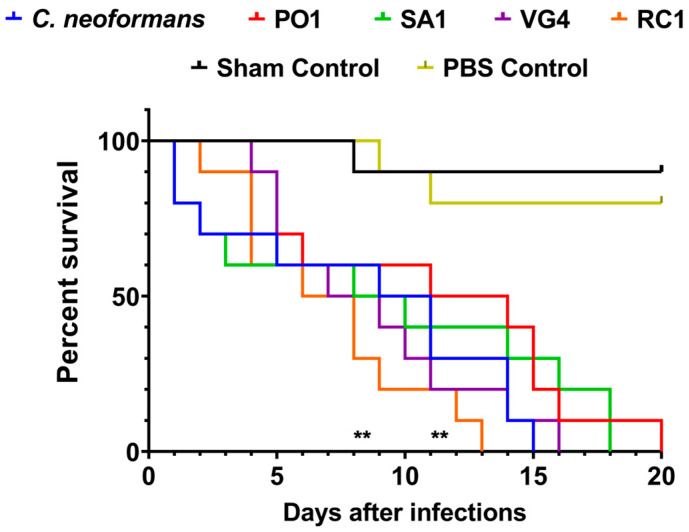
Survival analysis in *T. molitor*. Animals were infected with *R. mucilaginosa* strains and the reference strain of *C. neoformans*. Sham animals and animals inoculated with PBS served as controls. ** (*p*-value < 0.001, determined by Cox test).

An invertebrate survival experiment was conducted using *T. molitor* insects to evaluate the virulence potential of the studied fungi (Figure 8). Through the survival curve analysis employed in this study, it was possible to observe that two strains of *R. mucilaginosa* (RC1 and VG4) and the strain of *C. neoformans* (H99) exhibited higher pathogenicity, resulting in significantly shorter survival times compared to the control groups. This result was considered statistically significant, with a *p*-value of <0.001 by the *t*-test. Conversely, sham control animals (those not inoculated or manipulated) and animals inoculated with a PBS solution displayed a longer lifespan and normal development into adulthood (Figure 8).

## 4. Discussion

Over the past decade, the emergence of new fungal infections has garnered increasing attention in the field of healthcare and epidemiology. Among them, *Rhodotorula* infections have gained prominence as a noteworthy example. These infections, caused by various species within the *Rhodotorula* genus, have posed unique challenges for healthcare providers and have necessitated a deeper understanding of their epidemiology, clinical manifestations, and treatment. The emergence of *Rhodotorula* infections underscores the need for heightened surveillance, research, and improved diagnostic tools. Healthcare providers must be vigilant in recognizing these infections, especially in vulnerable patient populations, and adjusting treatment strategies as necessary due to the challenges posed by antifungal resistance [8,9,13].

The results presented in this study yield significant insights into the comparative analysis of two yeast species, *C. neoformans* and *R. mucilaginosa*, with a particular emphasis on their polysaccharide capsules and various factors related to virulence. Our results support previous findings regarding the presence of a polysaccharide capsule around *R. mucilaginosa*, analogous to the capsule found in *C. neoformans*. This finding is of particular importance due to the capsule’s critical role as a virulence factor in *C. neoformans* [16,54]. In this species, the capsule plays a vital role in pathogenicity by assisting the fungus in evading the host’s immune system and establishing infections. The confirmation of a similar capsule in *R. mucilaginosa* suggests that the latter may also have similar virulence mechanisms or share characteristics with *C. neoformans* that warrant further investigation. Moreover, the study reveals that the capsule of *C. neoformans* is significantly larger than that of *R. mucilaginosa*. This observation suggests that *C. neoformans* may harbor a greater potential for virulence, as the capsule is considered a major virulence factor in the *Cryptococcus* genus. This may explain, at least in part, why cryptococcal infections are more common than those caused by the *Rhodotorula* genus. This finding also aligns with prior research, reinforcing the pivotal role of capsule size in determining virulence [50].

In addition to differences in capsule size, the study identifies statistically significant variations in cell body size. And from these results, we can note that the *C. neoformans* yeasts are larger than those from the *R. mucilaginosa* strains. These size disparities have implications for fungal virulence, influencing processes such as cell migration, phagocytosis, and resistance to stress and antifungal agents. Furthermore, the Zeta potential analysis reveals distinctions in surface charge stability between the two species. Specifically, *C. neoformans* exhibits a more electronegative Zeta potential compared to *R. mucilaginosa*, which corroborates the study by Pontes and Frases (2015), which emphasizes that Zeta potential measurements can quickly estimate the relative differences in composition between ionic polymers [50]. This difference can be attributed to the presence of glucuronic acid, a component associated with higher electronegativity in *Cryptococcus* spp., suggesting a potential link between electronegativity and virulence [55]. The conductance analysis demonstrates that *R. mucilaginosa* strains exhibit higher conductance than *C. neoformans*. This indicates an increase in ionic charges in *R. mucilaginosa*, potentially linked to differences in cell wall composition and surface properties. Additionally, this study delves into the intensity and effective diameter of secreted polysaccharides. *C. neoformans* produces polysaccharides with greater intensity and larger effective diameter, potentially enhancing resistance to stress and antifungal agents. This underscores the role of polysaccharides in fungal virulence [56,57,58].

The immunolabeling experiments suggest potential differences in the architecture or specific epitopes of the capsule component between the two species. This study also reveals unexpected cross-reactivity between *R. mucilaginosa* and the *C. neoformans* antigen immunochromatographic lateral flow assay, underscoring the need for more reliable diagnostic tests [59]. It is important to highlight that the manufacturer’s documentation for the rapid test does not predict cross-reactivity with *R. mucilaginosa*. These results highlight the potential for variations in capsule architecture and epitopes between fungal species, and the need for reliable tests to ensure proper diagnosis and appropriate patient management.

The results obtained *in vitro* and *in vivo* have relevant implications for research and public health, as they can assist in better diagnosis and treatment of affected individuals. Through the survival curve analysis used in this study, it was possible to observe that the *R. mucilaginosa* strains RC1 and VG4 and the *C. neoformans* strain H99 showed greater pathogenicity, even though the *Rhodotorula* strains presented a smaller capsule size and cell body in relation to *Cryptococcus*, and SA1, RC1, and VG4 were less electronegative in relation to *Cryptococcus*; however, the survival curve was statistically significant, leading to the death of the animals in a significantly shorter time compared to the control groups, corroborating the results of the studies by Jarros et al. (2022) [24]. On the other hand, sham control animals (which were not inoculated or manipulated) and animals that were inoculated with PBS solution had a longer lifespan and normal development until adulthood. Considering that these animals were kept in similar environmental conditions such as temperature, humidity, and availability of food and water, it was concluded that fungal infection was a determining factor in insect mortality. This highlights the opportunistic pathogenic nature of *R. mucilaginosa* and its potential to cause diseases resembling cryptococcosis. Moreover, the survival curve analysis demonstrates the higher pathogenicity of both *R. mucilaginosa* and *C. neoformans* strains, leading to the significantly shorter survival of animals compared to control groups. These findings suggest the clinical relevance of these results and their potential implications for patient outcomes.

## 5. Conclusions

This work corroborates the study by Nazari et al. (2022) which draws attention to the importance of considering emerging and rare fungi as a differential diagnosis of COVID-19 complications [9], as well as for immunocompromised, bedridden patients, and those with some other risk factor.

This comprehensive study offers a detailed comparison of virulence factors between *C. neoformans* and *R. mucilaginosa*, emphasizing the significance of comprehending capsule properties, cell size, surface charge, and polysaccharide composition in the context of fungal virulence. Moreover, the results of this study carry substantial implications for diagnostic testing. They underscore the critical need for more precise tests capable of distinguishing between these closely related yeast species. Notably, the rapid cryptococcal antigen lateral flow assay (CrAg-LFA) test may exhibit unexpected cross-reactivity with *R. mucilaginosa*, contrary to the manufacturer’s claims. This misdiagnosis risk can have severe consequences, including the worsening of a patient’s clinical condition and, in severe cases, leading to fatality. This concern is exacerbated by the ineffectiveness of fluconazole treatment for *Rhodotorula* infections, as this genus is resistant to this antifungal.

The findings from this study significantly contribute to a deeper understanding of the biological and pathological aspects of the yeast species under investigation. They furnish invaluable information for the accurate identification of fungal infections and the judicious selection of appropriate antifungal treatments. Furthermore, the study highlights the opportunistic pathogenic potential of *R. mucilaginosa*, even in immunocompetent hosts. This underscores the need for diligent clinical considerations in the diagnosis and treatment of fungal infections. It calls for the implementation of effective control and prevention measures to mitigate the risks of cross-reactivity in diagnostic tests, diagnostic errors, and the subsequent deterioration of patient prognoses.

In essence, this study provides a critical foundation for the development of more accurate diagnostic tools and treatment strategies, aiming to improve patient outcomes and safeguard public health.

## Data Availability

Data are contained within the article.
